# Aerosol Transport Modeling: The Key Link Between Lung Infections of Individuals and Populations

**DOI:** 10.3389/fphys.2022.923945

**Published:** 2022-06-20

**Authors:** Chantal Darquenne, Azadeh A.T. Borojeni, Mitchel J. Colebank, M. Gregory Forest, Balázs G. Madas, Merryn Tawhai, Yi Jiang

**Affiliations:** ^1^ Department of Medicine, University of California, San Diego, San Diego, CA, United States; ^2^ Edwards Lifesciences Foundation Cardiovascular Innovation and Research Center and Department of Biomedical Engineering, University of California, Irvine, Irvine, CA, United States; ^3^ Departments of Mathematics, Applied Physical Sciences, and Biomedical Engineering, University of North Carolina at Chapel Hill, Chapel Hill, NC, United States; ^4^ Environmental Physics Department, Centre for Energy Research, Budapest, Hungary; ^5^ Auckland Bioengineering Institute, University of Auckland, Auckland, New Zealand; ^6^ Department of Mathematics and Statistics, Georgia State University, Atlanta, GA, United States

**Keywords:** respiratory droplets, aerosol deposition, mucociliary clearance, respiratory tract infection, public health

## Abstract

The recent COVID-19 pandemic has propelled the field of aerosol science to the forefront, particularly the central role of virus-laden respiratory droplets and aerosols. The pandemic has also highlighted the critical need, and value for, *an information bridge between epidemiological models* (that inform policymakers to develop public health responses) *and within-host models* (that inform the public and health care providers how individuals develop respiratory infections). Here, we review existing data and models of generation of respiratory droplets and aerosols, their exhalation and inhalation, and the fate of infectious droplet transport and deposition throughout the respiratory tract. We then articulate how aerosol transport modeling can serve as a bridge between and guide calibration of within-host and epidemiological models, forming a comprehensive tool to formulate and test hypotheses about respiratory tract exposure and infection within and between individuals.

## 1 Introduction

In the current environment of rising concerns over emerging infectious diseases, mathematical models of disease transmission have been used to understand how infections spread within and between individuals (hosts), thereby informing actionable tools for prevention and progression of infection, both in clinical practice and in public health policy decisions and communications. Two primary classes of disease transmission models are between-host and within-host models. The former separates populations into homogeneous compartments such as susceptible, infectious, and recovered subgroups (the SIR model) to study disease transmission across a population. The latter models focus on viral infection, replication, and transmission within the host, typically starting from a prescribed location of viral exposure and cell infection, then modeling the competition between progression of infected cells and viral replication versus clearance of infection and viruses by various innate, immune, or medical interventions.

Both classes of models have enjoyed significant historical and present success. *Epidemiological or between-host models*, including mechanistic SIR-type transmission models and various statistical models, have been widely applied to all conceivable infectious diseases. They provide qualitative and quantitative understanding of spread of disease in a given population, in terms of transmission history and prognoses of future spread or decline. At the onset of the COVID-19 pandemic, epidemiological models were rapidly adapted to emerging public health data and provided valuable support for policymakers to develop public health responses. For example, the Center for Disease Control and Prevention (CDC) continues to use a large and growing list of such models (see https://github.com/cdcepi/COVID-19-Forecasts/blob/master/COVID-19_Forecast_Model_Descriptions.md) to provide forecasts of new COVID-19 cases, hospitalizations and deaths (https://www.cdc.gov/coronavirus/2019-ncov/science/forecasting/mathematical-modeling.html). *Within-host models* often use a structure similar to susceptible-infectious (SI) epidemic models, with or without the protective role of immune response. In respiratory infections, additional protective roles of mucosal barriers and antibodies throughout the respiratory tract may also be considered. Within-host models describe organ-specific interactions between pathogens and target cells, focusing on cell-to-cell transmission in place of host-to-host. The most notable success stories are for HIV, hepatitis B, hepatitis C, and influenza viruses (Dahari et al., 2005; Hews et al., 2010; Pawelek et al., 2012; Perelson and Ribeiro, 2013; Wessler et al., 2016; Newby et al., 2017) where within-host models successfully predicted the outcome of different treatment strategies, e.g., inhibited viral mobility in mucus vs. inhibited replication vs. increased viral clearance vs. a combination. They, too, have been quickly adapted to model SARS-CoV-2 dynamics to offer insights into the pathogenesis and treatment of COVID-19 (Gonçalves et al., 2020; Kim et al., 2021; Perelson and Ke, 2021; Chen et al., 2022).

**FIGURE 1:**
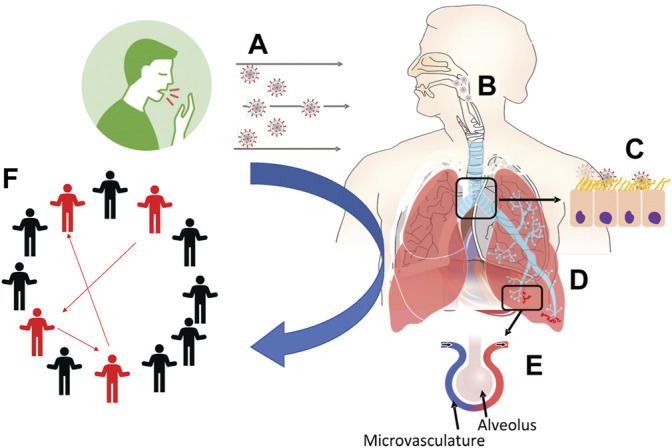
Schematic outline of the production, transport and fate of respiratory droplets among individuals as addressed in this article. **(A)** Exhaled breath and aerosol dynamics (Section 2). **(B)** Fate of inhaled aerosols in the respiratory system (Section 3). **(C)** Fate of inhaled infectious aerosols deposited at the air-airway surface liquid interface (Section 4). **(D)** Particle distribution effect on viral binding, importance of local dose versus averages over the whole lung (Section 5). **(E)** Endothelial dysfunction and pulmonary circulatory interactions with SARS-CoV-2 (Section 6). **(F)** Linking personalized models to population models (Section 7).

However, there is a glaring gap in information and knowledge sharing of viral transmission dynamics *between* these two classes of models. Despite the vast difference in the spatial and temporal scales of disease exposure and progression within a population and within an individual’s respiratory tract, an important opportunity exists to leverage insights and knowledge from between-host and within-host models. Filling information gaps between these classes of models with model-centric lessons learned can address fundamental questions (Figure 1):

1. When infectious viruses preside in the human airway, how and from where in the respiratory tract (RT) can they be transmitted to the environment? Given the viral titer and specific RT location, what activities (normal breathing, strenuous exercise, coughing, sneezing, speaking, singing, eating) rupture droplets or aerosols containing how many infectious virions that are exhaled or propelled into the surrounding airspace? How do mask pore size and thickness alter these outcomes? (Section 2).
2. Where do inhaled aerosols or droplets deposit in the RT? Is there a distribution of deposition location versus vehicle size and activity level of the individual? (Section 3).
3. How does the host mucociliary clearance process, which undergoes extreme transport gradients from the upper to lower RT, influence (i) the likelihood of infection for a given inhaled exposure, (ii) the rate of spread of infection, and (iii) the growth of the viral load? How quickly do infected cells replicate, how many infectious viruses are replicated, at what rate and for how long, and how do variants affect the answers? (Section 4).
4. How does infection spread: locally (cell-to-cell), semi-locally (by infected cells shedding virions into the airway surface liquid and infect non-nearest neighbor cells), and distally (between the upper and lower RT)? (Sections 4, 5).
5. How does microvascular dysfunction and pulmonary circulation interact with air flow dynamics to impact the progression of infection? (Section 6).
6. How can “personalized” models of within-host transmission be used to inform population scale disease transmssion, where age, BMI and many other possible factors can be incorporated? (Section 7).

Answers to these questions are key to assess the effectiveness of preventive and treatment measures and for leveraging information between within-host and epidemiological models. In the next sections, we describe some of the existing data and models that can facilitate bridging these gaps in knowledge and understanding, and explain why answers to certain questions are critical determinants of both outcomes and public health recommendations.

The multiscale modeling and viral pandemics working group of the Interagency Modeling and Analysis Group Multiscale Modeling (IMAG/MSM) Consortium (https://www.imagwiki.nibib.nih.gov/working-groups/multiscale-modeling-and-viral-pandemics) brought together diverse expertise that inspired the conception of this work in an effort to highlight how aerosol transport modeling can address some of the challenges of coupling epidemiological and within-host models and to raise the need for action into creating a comprehensive modeling platform for viral transmission from an individual to the population.

## 2 Exhaled Breath and Aerosol Dynamics

The ongoing COVID-19 pandemic has propelled the field of aerosol science to the forefront, in particular the topic of the production of respiratory droplets and the role they play in the transmission of viruses. In this article, we define *aerosols* as *any mixture of gas, liquid, and the particles suspended within.* Particles can be solid such as mineral dust, absent of liquid, or aerosols may consist of liquid droplets of diverse sizes such as in exhaled breath or cough or in a medical spray. Aerosol particles typically range from a few nanometers to >100 µm in diameter (Hinds, 1999). Their behavior is highly dependent upon their size and composition, but also upon the environment (e.g., relative humidity, ventilation airflow properties). Although 5 µm is often used to distinguish aerosols from droplets, the size distinction between aerosols and droplets should be ∼100 μm, which denotes the largest particle size that can remain suspended in still air for more than 5 s from a height of 1.5 m (Wang et al., 2021).

Respiratory droplets and aerosols are generated both in the deep lung and in the upper airway (larynx and/or oral/nasal passages). Their size, number, and composition are linked to the generation mechanism, site of generation and activity, i.e., breathing, singing, vocalizing, sneezing, or coughing (Table 1). Breathing alone produces sub-micron aerosols that originate from closing and opening of airways in the deep lung even in healthy subjects (Johnson and Morawska, 2009; Holmgren et al., 2010; Scheuch, 2020). As the diameters of the small airways decrease during exhalation, their fluid lining can bridge the airway lumen to form an occluding film. These liquid films rupture during the following inhalation, generating an aerosol that travels mouthwards during exhalation, and into the environment. Production of respiratory aerosols via this mechanism increases as breathing deepens (e.g., exercise); deeper exhalation further reduces the diameter of small bronchioles in the deep lung, increasing the number of bronchiolar membranes collapsing onto airway surface liquid films. The average size of deep-lung aerosols that are produced during breathing has been reported to be 0.3–0.7 µm.

**TABLE 1:** Exhaled aerosols generated from human respiratory activities.

	Breathing	Coughing	Sneezing	Speaking/singing
Origin	Small airways	Mouth, larynx, large airways	Nose, large airways	Vocal cords, oral passages
Mechanism	Film bursting	Expulsion of compressed air, turbulent flow in upper and large airways		Fluid filaments (vocal cords), fluid film (mouth)
Size range	0.01—2 µm (mean: 0.3–0.7 µm)	<1 µm—>100 µm		1–5 µm (vocal cords) < 1—100 µm (mouth)

Sneezing and coughing are initiated by closure of the glottis and contraction of the abdominal muscles, which results in a build-up of air pressure in the lungs. Sudden opening of the glottis causes the expulsion of compressed air at velocities that generate turbulent flows in the central and upper airways. The interaction between turbulent flow and fluid lining the airways results in production of aerosols and droplets with diameters ranging between <0.6 µm and >100 µm (Stadnytskyi et al., 2021). Finally, particle production during speaking and singing results from the vibration of the vocal cords and from the rupture of liquid films in the mouth.

Unlike modeling of the fate of inhaled aerosols, which has been extensively developed as described below, modeling the *formation* of respiratory aerosols is a much less studied area. This is largely due to the *unresolved nonlinear viscoelastic nature of airway mucus and nasopharyngeal liquids* that prevents faithful predictions of droplet formation and size distributions (Levy et al., 2014). To date, there is very little mechanistic understanding from first principles of the rupture of viscoelastic liquids from high Reynolds number airflow-generated shear stress or from closing and opening of wet membranes. A recent review (Mittal et al., 2020) identified the following *viscous fluid instabilities* to be relevant to respiratory droplet formation from air-flow generated shear stress: surface-tension-driven Rayleigh–Plateau instability (Eggers, 1997; Lin and Reitz, 1998; Romanò et al., 2019), shear-driven Kelvin–Helmholtz instability (Kataoka et al., 1983; Scardovelli and Zaleski, 1999) and acceleration-driven Rayleigh–Taylor instability (Joseph et al., 2002; Halpern and Grotberg, 2003) with the latter particularly important for coughing and sneezing. Similarly, rupture of a fluid meniscus generated by closing and opening of wet membranes is difficult to predict given the major role of moving boundaries, the large range of length and time scales implicated in this phenomenon, and especially the unresolved non-Newtonian properties of the fluids involved, i.e., mucus in the small airways and vocal cords, and saliva in the mouth, tongue, and lips. Recent progress on wet membrane rupture of viscous fluids, experimental and computational, has been described by Bourouiba (2021) and Abkarian et al. (2020).

Independent of their site of origin, respiratory aerosols all start out as liquid droplets with a 95–99% aqueous fraction. Once exhaled, they rapidly shrink through evaporation to reach an equilibrium with the water vapor content of the ambient air. This process usually takes only a few seconds and is most relevant for droplets with initial diameter <20 µm as these particles achieve equilibrium before settling or impacting on surfaces (Niazi et al., 2021). In still air, a 0.1 µm particle emitted by a 1.7 m tall standing individual will settle on the ground in ∼24 days, a 1 µm particle in ∼14 h, a 10 µm particle in ∼10 min, and a 100 µm particle in less than 6 s. The size of SARS-CoV-2 viruses are reported to be ∼0.06–0.14 µm (Jin et al., 2020) or possibly smaller (Jiang et al., 2020). Thus, respiratory aerosols 0.1–1 µm in diameter containing SARS-CoV-2 virions easily remain suspended in the air for hours in poorly ventilated indoor environments.

## 3 The Fate of Inhaled Aerosols in the Respiratory System

While exposure to airborne particulates/droplets and its implication for human health have long been a major concern, the SARS-CoV2 pandemic has led to renewed interest in the fate of inhaled viral particles in the lungs and beyond. Aerosol deposition in the airway results from a complex interaction between the structure and dynamics of the inhaled-air velocity-field and the physical properties of the aerosol particles. A complete understanding of the deposition process requires understanding of both the particle and fluid dynamics of the respiratory airflow (Finlay, 2001).

The complex anatomy of the respiratory tract is an important factor affecting aerosol deposition. Inhaled aerosols first traverse the upper respiratory tract (nasal-pharyngeal and/or mouth cavities) before reaching the trachea and the intrathoracic region of the lung. In humans, the lower respiratory airways form a dichotomous tree where each airway gives rise to two child branches. On average, 23 branch divisions separate the terminal alveolar sacs from the trachea (Weibel et al., 2005). The first 15 generations are conducting airways, and the last eight to nine generations constitute the gas exchange region. The airways become shorter and narrower with each generation, however the increasing number of airways with each generation provides a substantially large increase in total airway cross-section and air-airway surface liquid interface. An important consequence for aerosol transport is that the increasing cross-sectional area results in a proportional decrease in airflow velocity and increase in aerosol residence time towards the lung periphery.

Deposition of inhaled particles occurs mainly via inertial impaction, gravitational sedimentation, and Brownian diffusion, and to a lesser extent by interception, turbulent mixing, and electrostatic precipitation (Darquenne, 2012). Briefly, inertial impaction results from the inability of particles to follow sudden changes in gas flow direction and is a primary mechanism for particles larger than 5 µm. As a velocity-dependent mechanism, deposition by inertial impaction occurs preferentially in the upper airway and the first few generations of intrathoracic airways where gas and particle velocity are high. Gravitational sedimentation results from settling of the particles under the action of gravity and significantly affects deposition of 1–8 µm diameter particles. Brownian diffusion is the dominant mechanism of deposition for particles less than 0.5 µm in diameter. Both gravitational sedimentation and Brownian diffusion are time-dependent mechanisms and as such are most efficient in the lung periphery where airspace size is small and residence time high.

The last few decades have seen major developments in modeling of aerosol transport in the lung. While early computational models of aerosol transport and deposition used simplified representations of the respiratory system (Landahl, 1950; Beeckmans, 1965), later models were based on a continuous description of aerosol transport in the lung (Taulbee and Yu, 1975; Darquenne and Paiva, 1994) where a one-dimensional (1D) convective-diffusive equation incorporating a term accounting for deposition was solved. These models have been successful in predicting overall deposition averages but failed to accurately predict local deposition. This is partly due to the single “typical” path nature of the lung model where deposition in each airway of a single path from the trachea to the alveolar sacs is multiplied by the number of airways in each generation to provide an estimate of total lung deposition. This approach implies that deposition in each airway of a given generation is similar and does not account for inhomogeneity in the branching pattern and/or subtended volume. As such, this type of model cannot incorporate heterogeneities in airway anatomy and tissue mechanics that are the hallmark of several lung diseases. The development of multiple-path models has partially addressed this limitation. For example, the “Multiple Path Particle Deposition” or MPPD model calculates particle deposition in all airways of the lung and provides lobar-specific and airway-specific information (Anjilvel and Asgharian, 1995). The MPPD model uses semi-empirical relationships in the upper airway and solves flow and deposition in the lower respiratory tract made of cylindrical airways. Other approaches include stochastic models accounting for the asymmetry of the airway tree (Koblinger and Hofmann, 1990) and more recently a model incorporating a Markov chain formulation of particle motion (Sonnenberg et al., 2020).

While 1D models can predict deposition for the entire respiratory system, they cannot describe site-specific deposition within individual airways or in specific locations in the lung. More recently, computational fluid dynamics (CFD) combined with automated reconstruction of lung airways from clinical imaging has been used to create highly realistic lung models in which aerosol transport and deposition can be predicted (Ma and Lutchen, 2009; Corley et al., 2012). CFD models utilize three-dimensional (3D) geometries in which detailed governing flow and particle transport equations are used to predict the spatial pattern of deposited particles. These models, however, are more difficult to implement than 1D models, require extensive computing resources and thus typically only focus on a specific region of the lung (Kleinstreuer and Zhang, 2003; Liu et al., 2007; Darquenne et al., 2009; Ma and Lutchen, 2009; Darquenne et al., 2011; Hofemeier and Sznitman, 2016; Haghnegahdar et al., 2019). Recently, multiscale strategies have been developed to link different models that apply to different lung regions to obtain a realistic subject-specific picture of the fate of inhaled aerosols. One approach has been to integrate distal lung mechanics through coupling of the 3D CFD model of the upper airway and large conducting airways with 0D or 1D models of the distal lung at each outlet (Kuprat et al., 2013; Kuprat et al., 2021). 0D models are represented by ordinary differential equations representing the compliant mechanics of the airways while 1D models can be represented by single or multiple path models. Promising preliminary results suggest that hybrid models, while still in early development, can accurately predict site- and region-specific deposition of aerosols. Such models can thus be an effective tool to explore and understand the connection between aerosolized viruses produced by a person with a respiratory tract infection and inhaled exposure. This between-host model understanding then informs the input conditions for within-host models of disease. And to complete the feedback loop, the within-host models inform viral loads and sites of infection that are the sources for exhaled aerosolized viruses. This combination of models will ultimately guide understanding and predictability of risk for individuals and populations.

## 4 The Fate of Inhaled Infectious Aerosols Deposited at the Air-Airway Surface Liquid Interface: Pre- and Post-Immunity Responses

A wide range of within-host models (Warrender et al., 2006; Mitchell et al., 2011; Pawelek et al., 2012; Levin et al., 2016; Quirouette et al., 2020; Sanyal, 2020; Kim et al., 2021; Leander et al., 2021) have been applied to viral diseases. The aim of all such models is to understand—physiologically and mechanistically—the fate within the human respiratory tract (RT) of exposure to inhaled aerosols containing infectious viruses. The diversity of models reflects the daunting complexity of this challenge. Every model includes compromises to reach a manageable description of the physiology of the respiratory tract, the viruses of interest and their transport properties, the kinetic rates and timescales of virus-cell interactions, infection and replication of daughter viruses, and diverse immune responses from macrophages, T cells, antibodies induced by infection, vaccination or drug delivery. Within-host models may be deterministic or stochastic, some track only the populations in time of model species while others resolve spatial spread in local regions or the entire respiratory tract.

The Forest group has constructed a modeling platform incorporating the first line of defense in the RT: mucociliary clearance (MCC) (Chen et al., 2022). Mucus lines the entire RT except the alveolar ducts and sacs and is propelled by carpets of coordinated beating cilia; the mucus barrier traps and transports inhaled insults toward the throat to be swallowed and cleared to the stomach. Inhaled aerosols deposit at either the air-mucus or air-alveolar fluid interface. To infect cells, a race ensues: virions must diffuse through the mucus barrier to encounter epithelial cells faster than the mucus escalator transports and clears the trapped cargo. Any virion that lands in the alveolar region will infect unless it is first captured by macrophages. When virions win the race, they infect cells and replicate infectious daughter virions. The predominant spread of virions and infection is via shedding of daughters into the airway surface liquid, not cell-to-cell infection. The baseline modeling platform predicts two outcome metrics from inhaled exposures assuming only MCC protection: the number and location in the RT of infectious virions and infected cells.

Next, the modeling platform superimposes second lines of defense, beginning with the immune system: macrophages, T cells, and antibodies (Ab) induced by vaccine or infection. Drug interventions are also incorporated: engineered monoclonal Ab, antiviral drugs, and mucolytic agents (e.g., to accelerate mucus transport or enhance binding affinities between Ab, virion spikes, and mucin polymers). The secondary platform orchestrates the evolution in an immense RT landscape of all molecular species (virions, Ab, drugs, mucus), their transport and mutual binding affinities, specificities of infectable cells, probabilities of infection per virion-cell encounter, daughter replication rates and duration by infected cells, macrophage- and T cell-induced knockdown in half-lives of virions and infected cells.

These comprehensive modeling elements are the result of a 20+year collaborative effort, the Virtual Lung Project (VLP) at the University of Carolina at Chapel Hill (UNC), integrating the knowledge and capabilities from across the medical, clinical, and basic sciences and applied mathematics. The VLP, and the Forest group in particular, were serendipitously poised to adapt the acquired modeling, experimental, and clinical capabilities in response to the COVID-19 pandemic.

**FIGURE 2:**
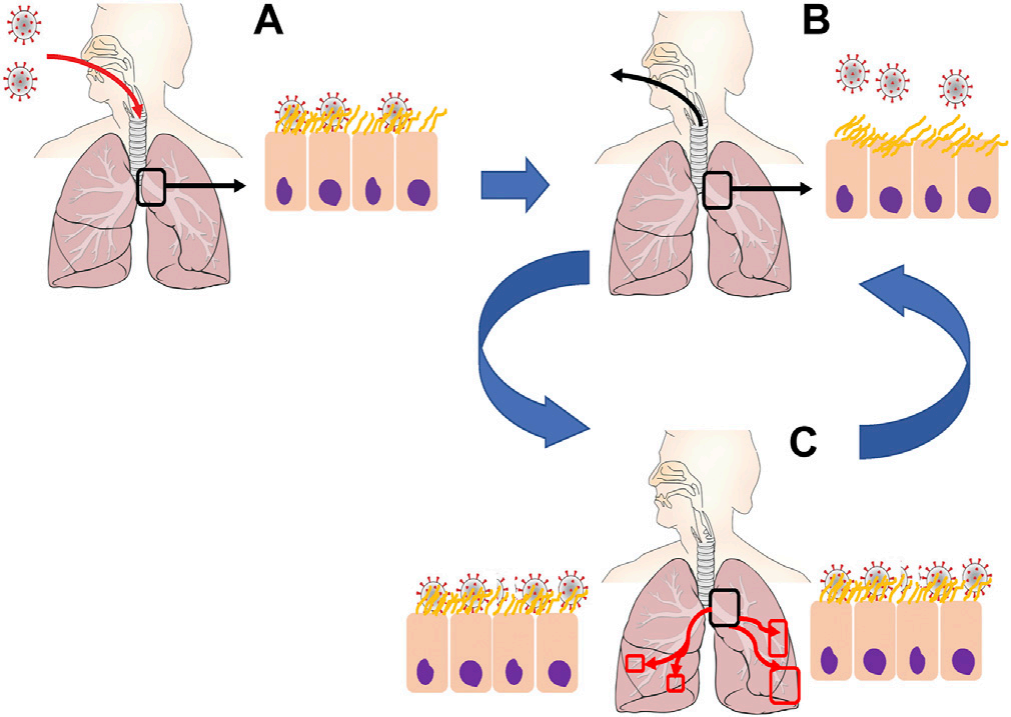
Proposed mechanisms of SARS-CoV-2 infection. **(A)** Initial inhalation of virions is captured by mucosal liquid lining within the upper respiratory tract or early generations of the bronchial tree **(B)** Human respiratory activity (e.g., talking, singing, screaming, coughing). causes closing and opening of mucosal membranes, rupturing fluid droplets containing infectious virions. **(C)** Microaspiration of oropharyngeal fluid and/or re-inhalation of aerosolized virions deposit into lower generations of the airway tree, allowing for replication and increased virus density throughout the deep lung.

The pre-immune response baseline model (Chen et al., 2022) focuses first on pre-vaccine clinical observations. It confirms the likelihood of a high-titer nasal infection from inhaled aerosols with SARS-CoV-2, while revealing an important insight: alveolar infection requires direct deposition of infectious seeds directly into the alveolar region, and furthermore, 10^6^ infectious seeds or more are needed to infect a significant (1% or more) proportion of the 140 m^2^ alveolar surface. These results are consistent with clinical observations that a large fraction of COVID-19 patients aspirate nasal boluses that drain and divide scores of times into the deep lung, effectively aerosolizing many thousands of infectious seeds for each 1 ml bolus (Figure 2).

The modeling platform is responsive to evolution in the pandemic. For example, it is possible to simulate variability in outcomes from exposures due to structural protein mutations of SARS-CoV-2 variants (Korber et al., 2020; Wölfel et al., 2020; Shen et al., 2021): the receptor binding domain (RBD) site, the N-terminal, and the cleavage site at the S1-S2 junction. The model proxies for these mutations are: probability of infection per encounter (RBD-ACE2 receptor binding affinity), length of time spanning virion-cell binding, cellular uptake, assembly, replication and shedding of daughters (the so-called eclipse phase, ∼ 12 h for the δ variant); and, replication rate and duration of infectious daughter viruses (1,000–2,000/day for 3–4 days for the δ variant). The model reveals strong sensitivities and dramatic differences in outcomes from nasal infections due to RBD-ACE2 binding affinity, shorter eclipse phase prior to infected cell replication, or more efficient replication of infectious daughters. The δ variant is reported to have stronger RBD-ACE2 binding affinity, so in lieu of the observed orders-of-magnitude higher titers on a compressed timescale, the model strongly points to a shorter eclipse phase due to the N-terminal or S1–S2 junction mutations (Pearson et al., 2022). Further insights to be gained from the post-immune response model include the variability in outcomes due to Ab from the different vaccines or infection, or therapeutic synthetic Ab. E.g., what are: the Ab titer per location in the RT, the Ab binding affinities to spike domains and to mucin polymers? The model generates these outcomes and differences if properties are known. One can either measure the properties to compare model predictions with clinical outcomes or infer what the properties are from clinical outcome data. Either way, mechanistic insights are gained for future pandemics or seasonal flu outbreaks. Furthermore, such insights guide therapeutic design. These examples illustrate the value of feedback between an accurate mechanistic model, clinical outcomes, and medical strategies.

## 5 Particle Distribution Effect on Viral Binding, Importance of Local Dose Versus Averages Over the Whole Lung

**FIGURE 3:**
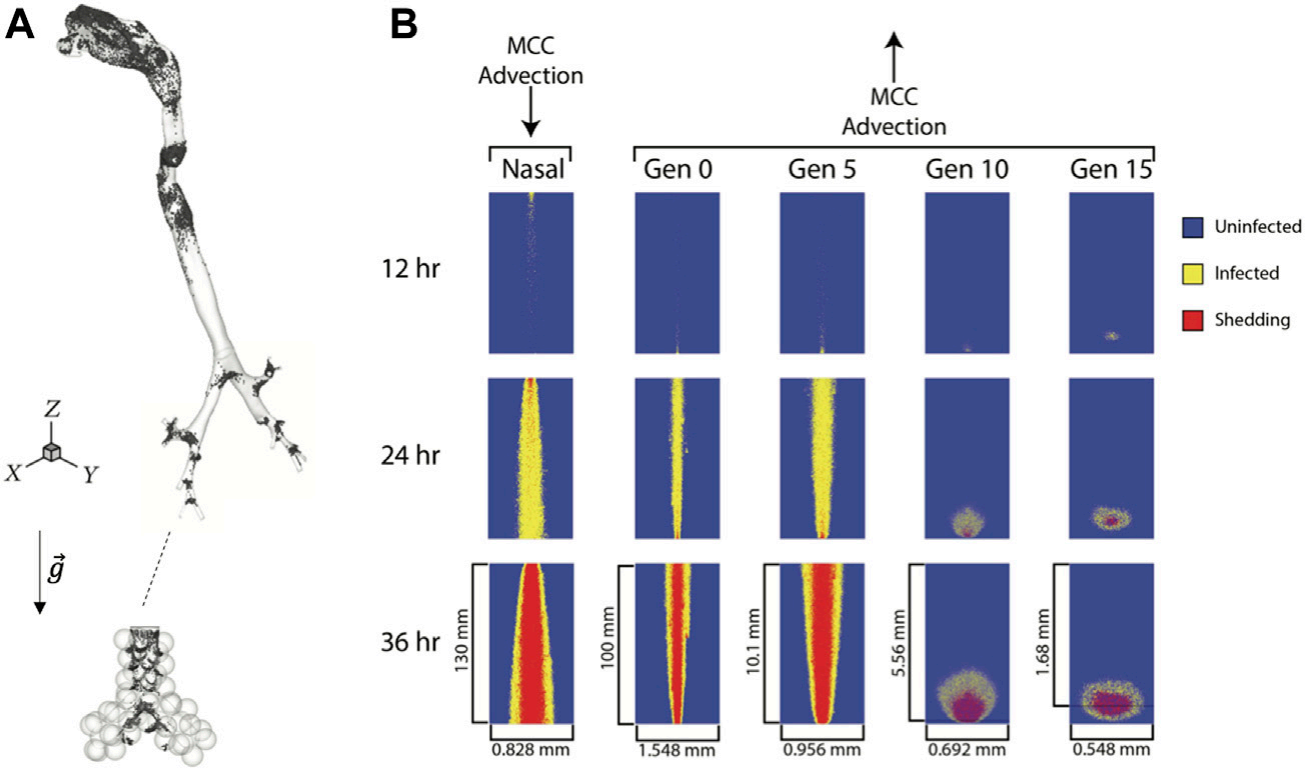
Viral particle deposition and spread of infected cells. **(A)** Deposition patterns of 1 µm particles inhaled at a flow rate of 0.5 L/s in a model of the human lung highlighting the heterogeneous nature of deposition patterns both in the conducting airways and in the alveolar region (not to scale), $\vec{g}$ : gravity vector (modified from (Li et al., 2014) and (Ma and Darquenne, 2011)). **(B)** Snapshots of the spread of infected epithelial cells from a single infected cell in the nasal passage, trachea (Gen 0), Gen 5, 10 and 15 over a period of 36 h. Note the horizontal and vertical scales for each column are specific to each generation (reproduced from (Chen et al., 2022) copyright 2022, with permission from Elsevier).

The deposition distribution of virions can play a crucial role in the development of an infectious disease (Madas et al., 2020). For COVID-19, infection of the upper airways can result in mild symptoms, whereas infection of the alveolar region can lead to life-threatening pneumonia. Aerosol transport through repeated bronchial airway bifurcations results in a highly heterogeneous deposition (Balashazy et al., 2003), and the initial spatial distribution of SARS-CoV-2 is similarly heterogeneous. Although mucociliary clearance smooths the degree of heterogeneity to some extent, its effectiveness is decreased by the low mucus velocity in the peak of the bronchial bifurcations, which are the most exposed parts of the lungs (Farkas, 2020). Hot spots of deposited particles at airway bifurcations occurs at all levels of the respiratory tract from the conducting airways down to the alveolar region of the lung (Figure 3A). Also, in the small airways and the lung periphery, sedimentation becomes a significant transport mechanism for micron-sized particles with deposition patterns being directly affected by the direction of gravity with respect to the airway and alveolar walls.

Heterogeneity can be characterized by the maximum particle enhancement factor, defined as the ratio of the maximum particle density to the average particle density over a given part of the lungs (Balashazy et al., 1999). For a simplified upper tracheobronchial model, it has been shown that deposition enhancement factors for microparticles ranged from 40 to 2,400 and for nanoparticles from 2 to 11 (Zhang et al., 2005). There is also experimental evidence for high particle concentrations at airway carinas (Churg and Vedal, 1996).

The higher local concentrations of SARS-CoV-2 in the airway branching areas may significantly enhance the infection probability of the airways. The infection of a cell requires interaction between a virion and one of the receptors. In some cases, however, it may be required that multiple virions reach the surface or the inside of the cells to start the replication process. In such cases, deposition hot spots may play a crucial role in COVID-19 progression. The importance of the minimal virion number can be illustrated as follows: let us assume for illustrative purpose that the probability that at least one virion reaches a receptor in the airways is 10^–3^. If a single interaction is enough for cellular entry, and cellular entry induces viral replication with 100% probability, then the infection probability is also 10^–3^. If two virions were required for cellular entry (and the cellular entry induces viral replication with 100% probability), and events that different virions interact with the same cell are independent of each other, then the infection probability would be 10^–3^ × 10^–3^ = 10^–6^.

While in most cases, a single virion can enter the cell without the need for other virions attaching to the same cell, enzymes can destroy the virion before it can start the replication process, and thus inhibit the infection. If multiple virions enter the cell around the same time, it may overload the enzymatic protection resulting in a non-linear relationship between infection probability and the number of virions interacting with the same cell. Therefore, the expected number of virions required to infect a cell will be higher than one even if there is a chance that a single virion can induce viral replication. Once infected, the spread of an infected cell will depend upon its location in the respiratory tract. In large airways, infected cells will show a strong mouthward spread due to mucus transport towards the pharynx while those in the small airways (generation 10 and beyond) tend to remain local due weaker mucus advection (Figure 3B) (Chen et al., 2022).

The importance of deposition hot spots is even stronger if it is accompanied by a heterogeneous distribution of target cells. SARS-CoV-2 shows a gradient infectivity from the proximal to distal respiratory tract (Hou et al., 2020). In addition, ciliated cells are the primary targets for SARS-CoV-2 infection (Hou et al., 2020), and there are more ciliated cells in the carina region than in distal airways. Local dose and presence of target cells strongly affect infection patterns in the lung. It is thus not surprising that autopsy studies of the lung of COVID-19 patients have shown patchy rather than uniform disease (Hou et al., 2020).

## 6 Endothelial Dysfunction and Pulmonary Circulatory Interactions With SARS-CoV-2

The airway system functions in parallel with the pulmonary circulation to reoxygenate blood. Briefly, the pulmonary circulation is a high flow/low pressure system, due in part to the usually high compliance of the pulmonary vascular wall. In a healthy cardio-pulmonary system, partially deoxygenated blood is ejected out the right ventricle at systolic pressure of ∼25 mmHg, which is much lower than the 120–130 mmHg systolic pressure of the left ventricle. Blood travels through the main and then left and right pulmonary arteries, and then through a rapidly branching tree to reach the pulmonary capillaries. The average 15 conducting airway generations in the human lung supply approximately 2^15^ acini. Each acinus has an average eight to nine generations of branching arterioles and venules that are connected by millions of capillaries comprising the lung’s gas exchange surface. The unique structure of the pulmonary capillaries as a network of very short segments covering the alveolar walls and surrounded on two sides by air, means that blood flow at this scale is more *sheet-like* than tube-like (Fung and Sobin, 1969; Fung and Sobin, 1972). It also means they are highly influenced by lung expansion or deformation and can become compressed as the alveolar wall is stretched.

A key biomarker of pulmonary circulatory coupling to the airway system is the ventilation ( $\dot{V}$ ) perfusion ( $\dot{Q}$ ) ratio, $\dot{V}/\dot{Q}$ . Ideally, at the whole lung scale the alveolar ventilation should be matched with cardiac output, i.e., $\dot{V}/\dot{Q}\approx 1$ . However, pulmonary hemodynamics and ventilation are influenced by gravity and the variable resistances of airway and vessel paths (West et al., 1964; Kang et al., 2018). Hence, $\dot{V}/\dot{Q}$ is spatially heterogenous, with a lung height dependence and considerable iso-gravitational heterogeneity (Mure et al., 2000): $\dot{V}/\dot{Q}\approx 0.6$ at the base of the lung, and $\dot{V}/\dot{Q}\approx 3.3$ in the apices (West et al., 1964). The two $\dot{V}/\dot{Q}$ ratio extremes are alveolar dead space ( $\dot{V}/\dot{Q}\to \infty$ ) and pulmonary shunt ( $\dot{V}/\dot{Q}=0$ ). The former occurs when blood flow is close to zero, and the latter when the supplying bronchiole is constricted or the tissue is too stiff to easily expand, leading to negligible ventilation and local alveolar hypoxia. SARS-CoV-2 promotes endothelial dysfunction, vascular leakage, and pulmonary microthrombi through inflammation, hypoxia, oxidative stress, and mitochondrial dysfunction (Potus et al., 2020). Blood viscosity is also often abnormally elevated (Wise, 2020). Pulmonary edema (e.g. in response to SARS-CoV-2) reduces the local tissue compliance and increases the thickness of the gas exchange barrier, leading to poorly ventilated tissue with low alveolar partial pressure of oxygen. Hypoxic pulmonary vasoconstriction (HPV) is a normal regulatory response to alveolar hypoxia that acts to redirect blood to more oxygen-rich alveoli. HPV occurs during the onset of SARS-CoV-2 (Karmouty-Quintana et al., 2020; Potus et al., 2020). Together these increase $\dot{V}$ (and hence $\dot{V}/\dot{Q}$ ) heterogeneity via dead space or high $\dot{V}/\dot{Q}$ distal to occluded regions, and low $\dot{V}/\dot{Q}$ in other regions that receive blood that is diverted away from poorly perfused tissue (Burrowes et al., 2011). Patients therefore have a combination of abnormal ventilation that leads to small blood vessel vasoconstriction, and abnormal hemodynamics because of vascular injury and increased blood viscosity. Whether this is sufficient to acutely elevate right ventricular pressure is not clear, and the effects of the virus on long-term pulmonary vascular function are still unknown. What is clear is that vascular-airway interactions are important for understanding symptoms and the progression of disease (Potus et al., 2020).

Computational modeling of blood flow through the pulmonary circulation has received less attention than models of airflow. Models tend to focus on understanding physiological interactions that determine $\dot{Q}$ distribution (Clark et al., 2011) or progression of vascular diseases (Clark et al., 2014; Qureshi et al., 2019; Colebank et al., 2021) with only a few e.g., (Fung and Sobin, 1972; Clark et al., 2010; Haber et al., 2013) having focused on pulmonary hemodynamics at the capillary level, and even fewer e.g., (Burrowes et al., 2011; Burrowes et al., 2014; Herrmann et al., 2020; Marquis et al., 2021) considering airway-circulatory interactions within the alveoli. For example, Burrowes et al. (2014) coupled a model of pulmonary circulatory dynamics with both ventilation and oxygen transfer models to investigate the effects of HPV in pulmonary embolism. Marquis et al. (2021) investigated HPV using a coupled mathematical model of pulmonary arteriolar and venular hemodynamics, ventilation, oxygen transport, and an empirical model of HPV. Herrmann et al. (2020) examined the effects of large perfusion defects to better understand early SARS-CoV-2 hypoxemia, concluding that hypoxemia during the onset of disease may be explained by a combination of alterations in HPV regulation, microthrombi-induced perfusion defects, or severe $\dot{V}/\dot{Q}$ mismatches. These approaches to understanding airway-circulatory coupling, largely through $\dot{V}/\dot{Q}$ , provide an excellent starting point for *in-silico* experiments of aerosol treatment for SARS-CoV-2.

Lung-vascular interaction at the microvascular level must be considered in the progression of SARS-CoV-2 and other cardiopulmonary diseases. While ACE2 receptors, the major binding site for SARS-CoV-2 in the alveolar epithelium, are understudied in the pulmonary vasculature (Stenmark et al., 2021), it has been hypothesized that pericytes, microvascular mural cells that regulate endothelial barrier function and inflammatory signaling, are damaged in response to SARS-CoV-2 binding in the alveolar epithelium (Cardot-Leccia et al., 2020; Stenmark et al., 2021). In contrast to pulmonary endothelial cells, pericytes are also known to express high levels of ACE2, and pericyte apoptosis has been reported in several SARS-CoV-2 cases (Cardot-Leccia et al., 2020). Regardless of the exact interaction at the vascular scale, pulmonary endothelial response to SARS-CoV-2 includes coagulation cascades, loss of vascular integrity, and increased production of reactive oxygen species (ROS) (Teuwen et al., 2020; Stenmark et al., 2021). Endothelial dysfunction is, thus, secondary to alveolar response due to aerosol deposition, but is probably a major contributor to the uncontrolled inflammatory response and cytokine storm often seen in those severely affected by SARS-CoV-2. The prevalent inflammatory response in combination with apparent micro thromboembolic clots and HPV in SARS-CoV-2 patients reflects the sensitivity of the cardiopulmonary system to the insult of the virus (Cardot-Leccia et al., 2020; Teuwen et al., 2020; Stenmark et al., 2021). This interplay encourages the development of more integrative computational models that account for pulmonary alveolar-endothelial interaction, which could provide a more precise mechanism for the transition from aerosol droplet inhalation to pulmonary capillary uptake and inflammatory response.

## 7 Personalized and Sub-Groups Specific Models

Age and BMI are associated with the risk of contracting COVID-19, and both are risk factors for developing severe disease (Davies et al., 2020; Zhang et al., 2020; Jung et al., 2021; Kompaniyets et al., 2021). The number of cases and the risk of developing severe disease increases with age, with a very low proportion of infections reported in children and much higher numbers and severe disease in older age (Davies et al., 2020). It has been estimated that susceptibility to infection in the <20 years age group is about half that of those aged >20 years (Davies et al., 2020). It is not clear whether children actually have lower susceptibility to infection, or whether they just experience milder or non-symptomatic infection. Conversely, subclinical infection in the elderly is rare. A study of unvaccinated COVID-positive patients in South Korea (prior to the availability of vaccines) found a greater risk of contracting COVID-19 in overweight and obese individuals (adjusted odds ratios of 1.12 and 1.26, respectively) (Jung et al., 2021). BMI has also been found to be associated with increase in ICU admission due to COVID-19, and BMI over 23 kg/m^2^ has been shown to be linearly associated with risk of severe COVID-19 leading to hospitalization and death (Gao et al., 2021). Underweight patients are also at risk of more severe disease (Ye et al., 2021). BMI, age, and ethnicity also have potentially interesting interactions, with higher adjusted hazard ratio for hospital admission for younger people (20–39 years) with BMI> 23 kg/m^2^ than those aged 80–100 years, and for Black people compared with White (Gao et al., 2021).

Understanding how age contributes to the transmission of disease will be important in understanding which public health interventions would be optimal for mitigating risk in social, educational, or workplace settings that have characteristic age groups. It is also important to understand how a restriction in lung function due to increased body weight contributes to BMI-related susceptibility and severity.

Age-dependent structure-function changes have implications for respiratory droplets generation and aerosol transport at multiple length scales. For example, it is likely that age-related changes to alveolar topology can influence alveolar deposition; and loss of lung tissue elastic recoil in the older lung increases ventilation heterogeneity *via* heterogeneous tissue expansion and airway narrowing. Large body mass that restricts lung expansion, and diseases that affect elasticity of the lung tissue or that narrow the airways will further impact on inhaled aerosol distribution and airway closure/reopening, hence the potential for respiratory droplet generation. Age, body mass distribution, and underlying respiratory disease are therefore all important factors in understanding the fate of inhaled aerosols and potential generation of droplets from the deep lung, in addition to representing risk factors for the severity of disease.

As described in previous sections, “personalized” models of portions of the airway tract (and pulmonary circulation) can be derived from medical imaging of individual subjects to act as geometric domains for simulation of transport and deposition. The airway or vascular geometry can be derived with reasonable accuracy for the largest airways and vessels. That is, to approximately generation 6–10 of airway for volumetric CT imaging from a healthy adult. When airways are narrowed—such as in older age or pathology—the number of airways that can be visualized reduces. Image-based models of the upper airways and vessels can be supplemented with representative volume-filling models of the many thousands of airways/vessels (Kitaoka et al., 1999; Tawhai et al., 2004; Burrowes et al., 2005) that cannot be visualized on imaging, to provide a 3D-1D domain that extends from the uppermost airway to the terminal bronchioles (Lin et al., 2009; Kuprat et al., 2021). Subject-specific lung motion can be coupled with the transport models to drive regional distribution of ventilation, using image registration between two or more image volumes (Yin et al., 2010) or using continuum models that simulate lung tissue deformation (Kang et al., 2018) during lung shape and postural change. Personalized models are limited by parameter uncertainty, for e.g., the exact distribution of airway dimensions and branching topology, and airway and lung tissue elasticity—amongst others. They are, however, useful for linking subject-specific distribution of pathology, e.g., emphysema with apical predominance, to transport and deposition. In contrast, subgroup models seek to include a range of anatomy and model parameterization that are representative of a subpopulation and physiologically reasonable.

To characterize aerosol inhalation across populations requires either a large model cohort that is representative of the population, or development of subgroup-specific models. A recent study shows how the former can be achieved with relatively limited imaging data: Xi et al. (2021) used a statistical shape modelling (SSM) approach to derive a population of 400 representative airway models as a virtual *in silico* cohort that represents variation in size and shape from a training cohort of only 40 subjects. The “virtual” models are generated by sampling the shape model mode weightings at specified intervals. Using this approach, any number of models can be generated that are not statistically different from the training cohort. SSMs have also been derived for the rib cage (Shi et al., 2014), lung shape (Torres-Tamayo et al., 2018; Osanlouy et al., 2020), and the paediatric airway tree (Humphries et al., 2016). Subgroup-specific cohorts can be developed in a similar way. That is, models that represent typical airway branching variants, and with airway and lung size and mechanics that depend appropriately on sex, age, smoking history, other exposures, and underlying respiratory disease. Ideally, shape models would be complemented with statistical distributions of tissue density and rib cage shape change between volumes. Subgroup models provide opportunity to address limitations in model parameterization, where—in the context of potentially important parameters for aerosol generation—individual small airway dimensions and compliances are not known. That is, subgroup models can be parameterized to statistical distributions of parameters that fit our understanding of physiology or pathophysiology, and their contribution to aerosol generation and transport evaluated through sensitivity analyses. Subgroup model output is then a mean and variability that reflects known inter-model differences, plus parameter uncertainty.

Subgroup-specific models also provide a potential link between detailed within-host models and epidemiological models. The within-host models can establish which structure-function features (including co-existing respiratory disease, age, BMI) are important for host infection, viral replication, and generation of infectious aerosol. Subgroup-specific models capture these features and how they relate to subgroups that are most prevalent in a population or that have vulnerabilities. Epidemiological models can introduce factors of mobility, socioeconomic impacts (e.g., housing quality and overcrowding, quality of nutrition), and access to primary care. Subgroup models thus provide a potential tool to examine both within-subject and between-subject phenomena within a single model framework. Environmental impacts can be translated to the within-host analyses, and conversely within-host details can be translated to epidemiological models to better-inform the reproduction rate for populations that comprise a given mix of subgroups.

## 8 Conclusion

A deeper understanding of aerosol dynamics poses as an enabling technology to tackle aerosol-based viral spread within both individuals and the population. We have highlighted the major mechanisms behind within-host modeling, specifically regarding aerosol inhalation, deposition profiles in the respiratory tract, the protective role of mucociliary clearance in the upper respiratory tract and large and medium-sized airways that propels the viral load toward the larynx to be swallowed, thus pointing to direct deposition of massive numbers of aerosols into the alveolar generations as the sole mechanism for deep lung infection, the pathway between respiratory infection and the pulmonary circulation, and broader impact on community-level disease spread. These models require individualized patient data for successful simulations, in contrast to epidemiological models which require less precise information regarding an individual’s physiological response to the disease. In addition to the challenge of modeling disease outbreaks at multiple spatial and temporal scales, it is difficult to gather data that accurately couples the epidemiological and within-host response to disease spread. Future protocols collecting data at all these levels will go a long way to building a comprehensive within- and between-host modeling toolkit that can be adapted and tailored to new waves of seasonal flu and cold viruses and future novel viruses like SARS-CoV-2 and its rapidly evolving mutant strains.

Nevertheless, the diverse modeling advances on host-to-host and within-host transmission of respiratory viral infection and disease are already impressive and have had impacts on decisions ranging from public health measures, clinical medicine, and drug development. The immediate challenge ahead is to create a comprehensive modeling platform that integrates within- and between-host physiology, viral exposure, infection, progression, and transmission -- the ultimate multiscale modeling challenge to create a “digital twin” for quantitatively accurate, virtual viral transmission within individuals and across the population.
